# CD40 signaling augments IL-10 expression and the tolerogenicity of IL-10-induced regulatory dendritic cells

**DOI:** 10.1371/journal.pone.0248290

**Published:** 2021-04-01

**Authors:** Wojciech Dawicki, Hui Huang, Yanna Ma, Jennifer Town, Xiaobei Zhang, Chris D. Rudulier, John R. Gordon

**Affiliations:** 1 Department of Medicine, University of Saskatchewan, Saskatoon, Saskatoon, Canada; 2 Department of Veterinary Microbiology, University of Saskatchewan, Saskatoon, Saskatoon, Canada; University of Manitoba, CANADA

## Abstract

CD40 expressed on stimulatory dendritic cells (DC) provides an important accessory signal for induction of effector T cell responses. It is also expressed at lower levels on regulatory DC (DCreg), but there is little evidence that CD40 signaling contributes to the tolerogenic activity of these cells. Indeed, CD40 silencing within DCreg has been reported to induce T cell tolerance in multiple disease models, suggesting that CD40 is superfluous to DC-induced tolerance. We critically assessed whether CD40 does have a role in tolerance induced by IL-10-differentiated DC (DC10) by using DC10 generating from the bone marrow of wild-type (w.t.) or CD40^-/-^ donor mice, or IL-10-complemented CD40^-/-^ DC10 to treat asthmatic mice. Wild-type DC10 ablated the OVA-asthma phenotype via induction of Foxp3^+^ Treg responses, but CD40^-/-^ DC10 had no discernible effects on primary facets of the phenotype (e.g., IL-5, IL-9, IL-13 levels, IgE & IgG1 antibodies; p>0.05) and were ≤40% effective in reversal of others. Foxp3^+^ T cells from the lungs of CD40^-/-^ DC10-treated mice expressed reduced levels of a panel of six Treg-specific activation markers relative to Treg from w.t. DC10-treated mice. Coculture with effector T cells from asthmatic mice induced a marked upregulation of cell surface CD40 on w.t. DC10. While untreated CD40^-/-^ and w.t. DC10 secreted equally low levels of IL-10, stimulation of w.t. DC10 with anti-CD40 for 72 h increased their expression of IL-10 by ≈250%, with no parallel induction of IL-12. Complementing IL-10 expression in CD40^-/-^ DC10 by IL-10 mRNA transfection fully restored the cells’ abilities to suppress the asthma phenotype. In summary, CD40 signaling in DC10 contributes importantly to their expression of IL-10 and to a robust induction of tolerance, including activation of induced Treg.

## Introduction

The context in which dendritic cells (DC) present antigens to T cells is important to their induction of effector versus regulatory T cell responses. When MHCII molecules on DC present processed antigen peptides to the T cell receptor (TCR), CD40 ligand (CD40L) on the T cell also engages the DC’s counterreceptor, CD40. That triggers a maturational change in the DC as a means of optimizing T cell:DC interactions. Thus, these DC upregulate their expression of MHCII, CD40 itself, TCR co-stimulatory molecules (e.g., CD80, CD86), as well as stimulatory cytokines such as IL-12 [1], each of which is seen by the T cell as an activation amplification signal [2]. This mutual feed-forward process is central to the DC’s successful induction of T cells as immunologic effector cells [2]. On the other hand, steady-state lung DC that present innocuous aeroallergens to T cells in their draining lymph node express low levels of CD40, MHCII, CD80 and CD86, and modest, but relatively higher levels of IL-10 than IL-12, and thereby induce regulatory T cell (Treg) responses [3]. Numerous reports have shown that the anergy-inducing properties of some regulatory DC (DCreg) are, at least in part, attributable to their expression of insufficient levels of MHCII, CD40 and co-stimulatory markers to support robust T cell activation (reviewed in ref. [2]).

It is also clear that IL-10 production by DCreg can contribute importantly to their tolerance responses but, as suggested above, more specifically that a combination of low level CD40 and costimulatory signaling and higher-level IL-10 expression fosters the differentiation of IL-10-expressing Treg (reviewed in ref. [4]). Despite their rather modest basal expression of IL-10, treatment with IL-10-differentiated DCreg (DC10) can reverse the asthma phenotype in mouse models [5, 6], while human DC10 similarly induce allergen- tolerance among CD4^+^ T cells of allergic asthma donors [7]. In both cases they do so by inducing CD25^lo^Foxp3^-^ effector T cells to transdifferentiate into CD25^hi^Foxp3^+^ Treg [7, 8]. Moreover, we know that IL-10 expression by DC10 is essential to their abilities to induce tolerance, such that IL-10 knock-out or IL-10-silenced, specific allergen-presenting DC10 have no impact on the disease phenotype in mouse models of asthma [9, 10].

As noted, CD40 signaling by immunostimulatory DC is integral their induction of effector T cell responses [11], but the role(s) for CD40 expressed by DCreg in induction of tolerance has received substantially less attention. It has been reported that CD40 signaling ablates induction of Treg by intestinal CD103^+^ (i.e., regulatory) DC, and that that leads to fatal colitis in a mouse model [12]. It has also been reported that immature DC that have not been exposed to CD40 agonists more efficiently induce Treg responses than do control DC that have been so exposed [13]. In addition, CD40-silenced immature DC have been noted to reverse the disease phenotype in mouse models of allergic rhinitis [14–16] and multiple sclerosis [17]. This suggests that CD40 signaling in DC is not only superfluous in tolerance responses, but that it may be inhibitory. Nevertheless, we hypothesized that CD40 signaling in DC10 does in fact contribute importantly to the regulatory activities of these cells and, given the critical role of IL-10 in the tolerogenicity of DC10 [9, 10], that CD40 signaling also regulates IL-10 expression in DC10. Herein we used wild-type (w.t.) and CD40^-/-^ DC10 to assess the role of CD40 in DC10 reversal of the asthma phenotype in OVA-asthmatic mice, complementing IL-10 expression in the CD40^-/-^ DC to assess whether IL-10 supplementation can over-ride a lack of CD40 signaling in these cells. Our data indicates that CD40 signaling substantially upregulates IL-10 expression in DC10 (by ≈250% over baseline levels), and that this contributes importantly to the cell’s tolerance-promoting activities, such that complementing IL-10 expression in these cells fully restores their abilities to induce asthma tolerance *in vivo*.

## Materials and methods

Animals. C57BL/6 and B6.129S2-*Cd40lg*^*tm1Imx*^/J (i.e., CD40^-/-^) mice, as well as ovalbumin (OVA)-specific T cell receptor-transgenic OT II mice were purchased from the Jackson Laboratory (Bar Harbor, ME); 6–18 wk-old female mice were used in all experiments. The animals were housed under specific pathogen-free conditions with food and water provided *ad libitum*. All experiments were approved by the Animal Research Ethics Board of the University of Saskatchewan (Protocol 19960112, approved 2009), in accord with the standards of the Canadian Council on Animal Care.

### Reagents

Antibodies to mouse CD4 (clone GK1.5), CD40 (hamster; clone HM40-3), CD25 (clone PC61.5), lymphocyte activation gene-3 (LAG3; clone eBioC9B7W), programmed cell death protein 1 (PD-1) (clone J43), glucocorticoid-induced TNFR-related protein (GITR; clone DTA-1), inducible T-cell costimulator (ICOS; clone 7E.17G9), cytotoxic T lymphocyte-associated protein 4 (CTLA-4; clone UC10-4B9), forkhead box P3 (FoxP3; clone FJK-16s), MHC II (clone M5/114.15.2), CD80 (clone 16-10A1), CD86 (clone GL1), OX40L (clone RM134L), CD40L (clone RM134L), ICOS ligand (ICOS L; clone HK5.3), PD-1 ligand 1 (PD-L1; clone MIH5), PD-1 ligand 2 (PD-L2; clone122) and isotype control antibodies, including the hamster IgG control (cat. No, 14-4888-81) for the anti-CD40 antibodies, were purchased from eBioscience (San Diego, CA). Anti-mouse IgG1 and IgE were from BD Biosciences (San Jose, CA). All paramagnetic sorting beads and columns were from Miltenyi Biotec (Auburn, CA). *Escherichia coli* 0127:B8 LPS and OVA were purchased from Sigma-Aldrich (Oakville, ON). GM-CSF and IL-10 were purchased from R&D Systems (Minneapolis, MN).

### Flow cytometry

Cells were washed three times with FACS buffer (0.01M azide, 2% FBS, PBS), incubated with blocking antibody for 10–20 min at 4°C, then labeled with surface marker-specific or isotype control fluorochrome-labeled antibodies at 4°C for an additional 20 min (or, in the case of CD40, for an additional 45 min). Cells to be probed also for intracellular markers were permeabilized (Fix/Perm Buffer; eBioscience, San Diego, CA) for 30 min, washed, incubated with blocking antibody in the same buffer for 10 min, and then labeled with marker-specific or isotype control fluorochrome-labeled antibodies for an additional 20 min, as reported [18]. Stained cells were washed three times and assessed with an EPICS XL flow cytometer (Beckman Coulter, Mississauga, ON) and the data analyzed using FlowJo version 8 software (Tree Star Inc., Ashland, OR).

### ELISA

Concentrations of IL-4, IL-5, IL-9, IL-10, IL-12p70, IL-13, and IFNγ in biological samples were determined by enzyme-linked immunosorbent assays (ELISA) using matched capture and detection antibodies and cytokine standards purchased from R&D Systems, as noted previously [9, 19]. The concentrations of allergen-specific IgG1 were determined by sandwich ELISA, using plate-bound OVA to capture the antibodies from serum and biotinylated anti-IgG for detection. The concentrations of allergen-specific IgE were also determined by sandwich ELISA, but therein we used plate-bound anti-IgE to capture the antibodies from serum and biotinylated OVA as a detection reagent [20]. Streptavidin-labelled alkaline phosphatase was used to visualize the captured biotinylated secondary antibodies or OVA. Serial dilutions of a pooled reference sera from mice twice immunized with allergen-alum was used as a standard, with arbitrarily assigned reference units set at 1000 for the undiluted reference sera [21].

### Generation of dendritic cells

All dendritic cells were differentiated from bone marrow precursors by plating ≈5x10^5^ bone marrow cells/ml in RPMI 1640 medium supplemented with 10% FBS, l-glutamine, antibiotics, and 20 ng/ml granulocyte-monocyte colony-stimulating factor (GM-CSF). Every 2–3 days ≈75% of the media in each culture was replaced. Tolerogenic DC10 were generated by decreasing the levels of GM-CSF to 7.5 ng/mL and adding IL-10 (10 ng/ml) from day 10 to 13 [19]. Stimulatory DC-LPS were differentiated for 7 days in 20 ng/ml GM-CSF and pulsed with 1 μg/ml LPS for the final 18 h of culture. Differentiated dendritic cells were pulsed with OVA (50 μg/ml) for the last 24 h of culture and then washed three times and counted before use (all DC populations routinely were ≥98% viable at the time of injection, as determined using trypan blue exclusion criteria). In experiments wherein the DC were stimulated with plate-bound anti-CD40 antibodies, the cells (10^6^/ml) were incubated in 24-well plates that had been precoated overnight with 10 μg/ml of anti-CD40 antibody [22].

### Asthma sensitization and regulatory DC treatment

C57BL/6 mice were injected i.p. with 200 μl of OVA-alum (2 μg OVA/mg alum) on days 0 and 14, and then exposed to 1% OVA aerosols for 20 min on days 28, 30 and 32. These asthmatic mice were then rested for 2 wk before i.p. injection of 1x10^6^ DC or saline, then 4 wk later they were challenged for 20 min with 1% OVA aerosols. We had previously titrated the numbers of DC10 required to optimally induce tolerance (Gordon, unpublished). We have also previously reported that i.p. delivery of DC10 was equally as effective as direct delivery into the airways, and that essentially full tolerance is achieved in the lungs of DC10-treated asthmatic mice at four wk post-treatment [19]. One day after this recall OVA challenge the animals’ airway hyperresponsiveness (AHR) was assessed. The next day the mice were sacrificed by exsanguination under surgical level methoxyfluorane anaesthesia. Airway cells and mediators were collected by bronchoalveolar lavage (BAL) for assessment of eosinophil and Th2 cytokine responses, while sera were collected for OVA-specific IgE and IgG1 assays. Cytocentrifuge preparations of the BAL fluid (BALF) cells were stained with Wright’s solution to generate differential cell counts [19, 20].

### Airway hyperresponsiveness

One day after OVA challenge the airway responses of the mice to carrier medium alone or increasing doses of nebulized methacholine (between 0.75 and 25 mg/ml) were assessed by head-out, whole body plethysmography [19, 20]. This parameter, gathered as running 1-s means of the air-flow at the 50% point in the expiratory cycle (Flow@50%TVe1), accurately reflects bronchiolar versus alveolar constriction or airway occlusion and accurately correlates with invasive measurements of AHR [23, 24].

### Transfection of DC

A cDNA coding for full-length IL-10 mRNA was cloned into pBluescript SK^+^ and transcribed using transcription and poly(A) tailing kits (mMESSAGE mMACHINE T7 T; Ambion, Foster City, CA) according to the supplier’s protocols. The IL-10 transcript was transfected into DC10, or the cells were sham-transfected using equivalent medium-loaded liposomes (TransMessenger Transfection Reagent; Qiagen, Toronto, ON). Liposomal loading was done at a 1:6 ratio of mRNA to transfection reagent, as per the supplier’s protocol. The optimal ratio of mRNA to liposomal reagent was determined in preliminary experiments by transfecting DC10 at ratios of 1:3, 1:6 or 1:12 and monitoring IL-10 mRNA levels by qRT-PCR 48h later. IL-10 protein expression was assessed at 24 and 48 hr post-transfection. The DC10 were used to treat asthmatic mice at 48 h post-transfection.

### DCreg suppression assays

#### Magnetic sorting of CD4^+^ cells

The lungs were harvested from OVA-asthmatic mice at 4 wk after their last allergen exposure. The tissues were dispersed mechanically, then passed through a 100 μm cell strainer (Corning Inc, Corning, NY) and the contaminating red blood cells lysed with hypotonic tris-ammonium chloride solution. CD4^+^ Teff cells were purified (≥92% CD4^+^; S1 Fig) using CD4-specific paramagnetic beads according to the supplier’s protocol.

#### DCreg suppression assays

We used *in vitro* T cell suppression assays to assess the function of our DCreg, as noted [7, 8]. Briefly, OVA-pulsed, irradiated DC10 (1000 rads) were titrated into round-bottom 96-well tissue culture plates with optimized numbers of irradiated OVA-presenting DC-LPS (4×10^3^ cells/well) and CD4^+^ Teff cells (1×10^5 T^ cells/well; 1:1, 1:3 or 1:9 DC10:T cell ratios) purified as noted above. We used ^3^H-thymidine uptake assays to assess T cell proliferation, as determined by liquid scintillation counting. We have previously reported that our Th2-phenotype Teff (i.e., CD4^+^CD25^lo^Foxp3^-^) cells are also CD44^hi^CD69^+^CD62L^lo^, and secrete IL-4, -5, -9 and -13 [25].

### Quantitative real-time PCR assays

RNA was isolated from cells using RNeasy (QIAGEN, ON) kits, while cDNAs were synthesized using qScript kits (Quanta Biosciences, MD), according to the supplier’s specifications. qRT-PCR reactions were performed using PerfCTa SYBR Green FastMix (Quanta Biosciences) with the appropriate primers in a C1000 Touch (BioRad, Mississauga, ON) thermocycler. Primers sequences were: IL10 forward AAGCCTTATCGGAAATGATCCA, reverse GCTCCACTGCCTTGCTCTTATT; IL12p35 forward GCCCTCCTAAACCACCTCAGT, reverse CAGGCAACTCTCGTTCTTGTGTA; and β-actin forward CCAGAGCAAGAGAGGCATCC, reverse CAACTGTCTCCATGTCGTCC. Data was analyzed using the software program CFX Manager (BioRad) and were normalized relative to β-actin mRNA levels.

### Statistical analyses

In experiments with only two study groups student’s t-tests were performed, while in all other experiments One-way ANOVA tests with Tukey’s *post-hoc* testing was used to compare pairs of experimental groups. All analyses were performed using GraphPad Prism software (La Jolla, CA). In all figures NS, *, **, or *** represent p values of >0.05, or ≤0.05, 0.01 or 0.001, respectively.

## Results

### CD40 is important to the regulatory activities of DC10

**Fig 1:**
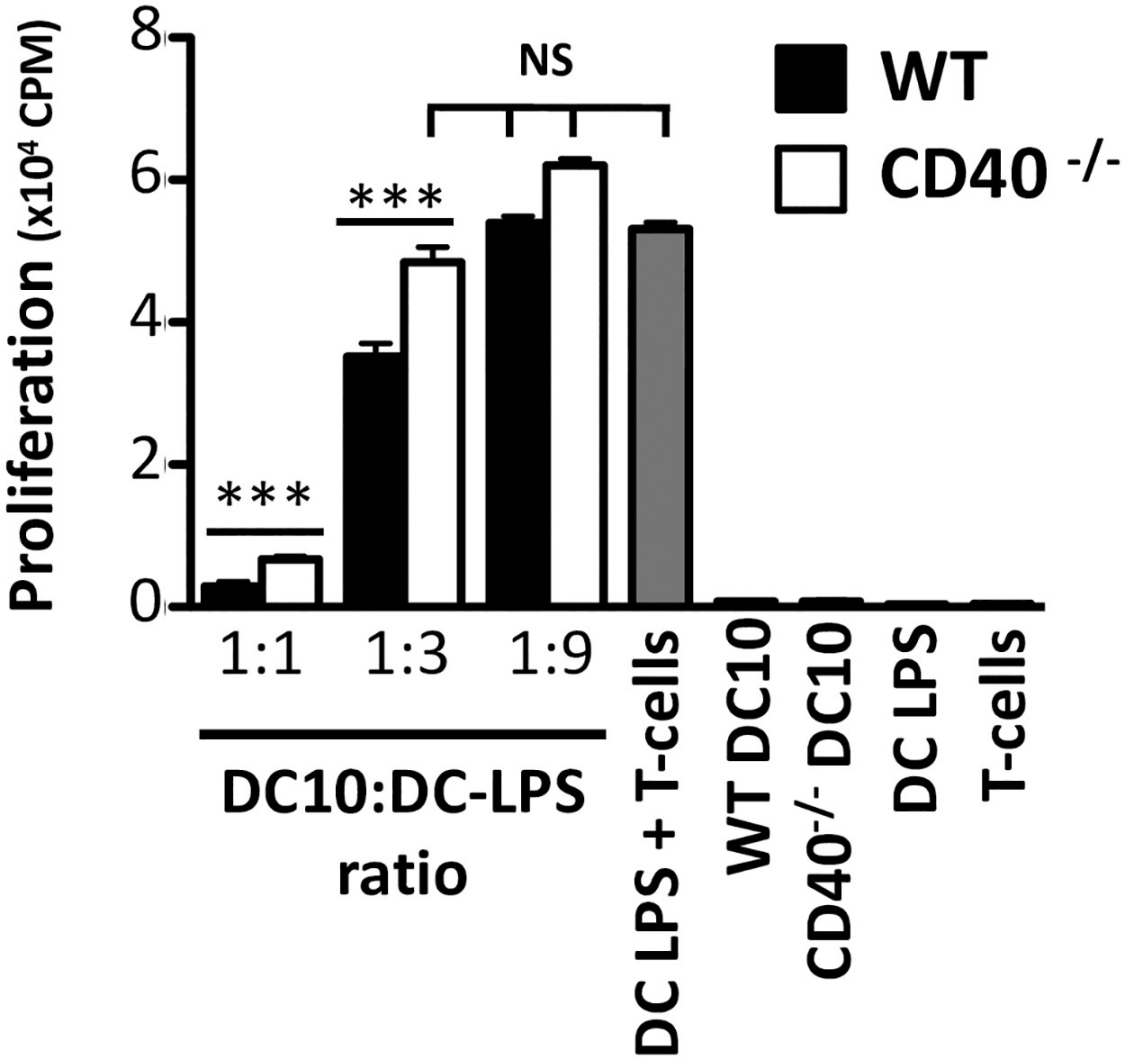
CD40^-/-^ DC10 have a reduced capacity to suppress T-cell proliferation relative to wild-type DC10. DC10 were differentiated from the bone marrow of wild-type (w.t.) or CD40 knock-out mice as noted in the Methods section and added at the indicated DC10:T-cell ratios to 3-day cultures of OVA-pulsed stimulatory DC-LPS and Teff cells (3x10^4^ DC-LPS, 2x10^5^ T cells) from the lungs of OVA-asthmatic mice. T cell proliferation was determined using standard ^3^H-thymidine-uptake assays. The data presented are representative of one of 3 independent experiments. NS or ***, p>0.05 or ≤0.001, respectively versus the DC-LPS/T cell control, as determined using one-way ANOVA tests with Tukey’s *post-hoc* testing.

We have reported that immature DC and IL-10-differentiated DC (DC10) express equivalent, but low levels of CD40 [5]. Others have reported that silencing or knock-out of CD40 in immature DC fosters strong, protective tolerance responses in mouse models of allergic disease [14, 15, 26] and multiple sclerosis [17]. Herein we assessed whether knocking out CD40 expression exclusively within DC10 would further augment their regulatory activities as determined *in vitro* using allergen-pulsed w.t. and CD40^-/-^ DC10. We assessed the expression levels of an array of cell surface proteins important for DC-T cell interactions, and found no differences in expression of MHCII, CD86, OX40L and CD40L, or the DCreg-associated markers ICOS-L, PD-L1 and PD-L2 between w.t. and CD40^-/-^ DC10 (S2 Fig). We then titrated these DC10 into cultures of immunostimulatory OVA-pulsed DC-LPS and CD4^+^ cells from the lungs of asthmatic OVA-TCR transgenic OTII mice, using the OTII T cell proliferative response as our readout. As expected, DC-LPS strongly activated the T cells in these cultures (Fig 1). Wild-type OVA-presenting DC10 significantly suppressed these DC-LPS-driven Th2 responses at DC10:DC-LPS ratios of 1:3 or 1:1 (both, p≤0.001; Fig 1). The CD40^-/-^ DC10, however, did not display discernible activity at ratios of 1:3 or 1:9 (p>0.05), although they were able to suppress Th2 responses when higher numbers of knock-out DC10 were added to the cultures (1:1 ratio; p≤0.001). All control cell populations (i.e., w.t. or CD40^-/-^ DC10, DC-LPS or T eff cells alone) yielded background signals in these assays (Fig 1).

**Fig 2:**
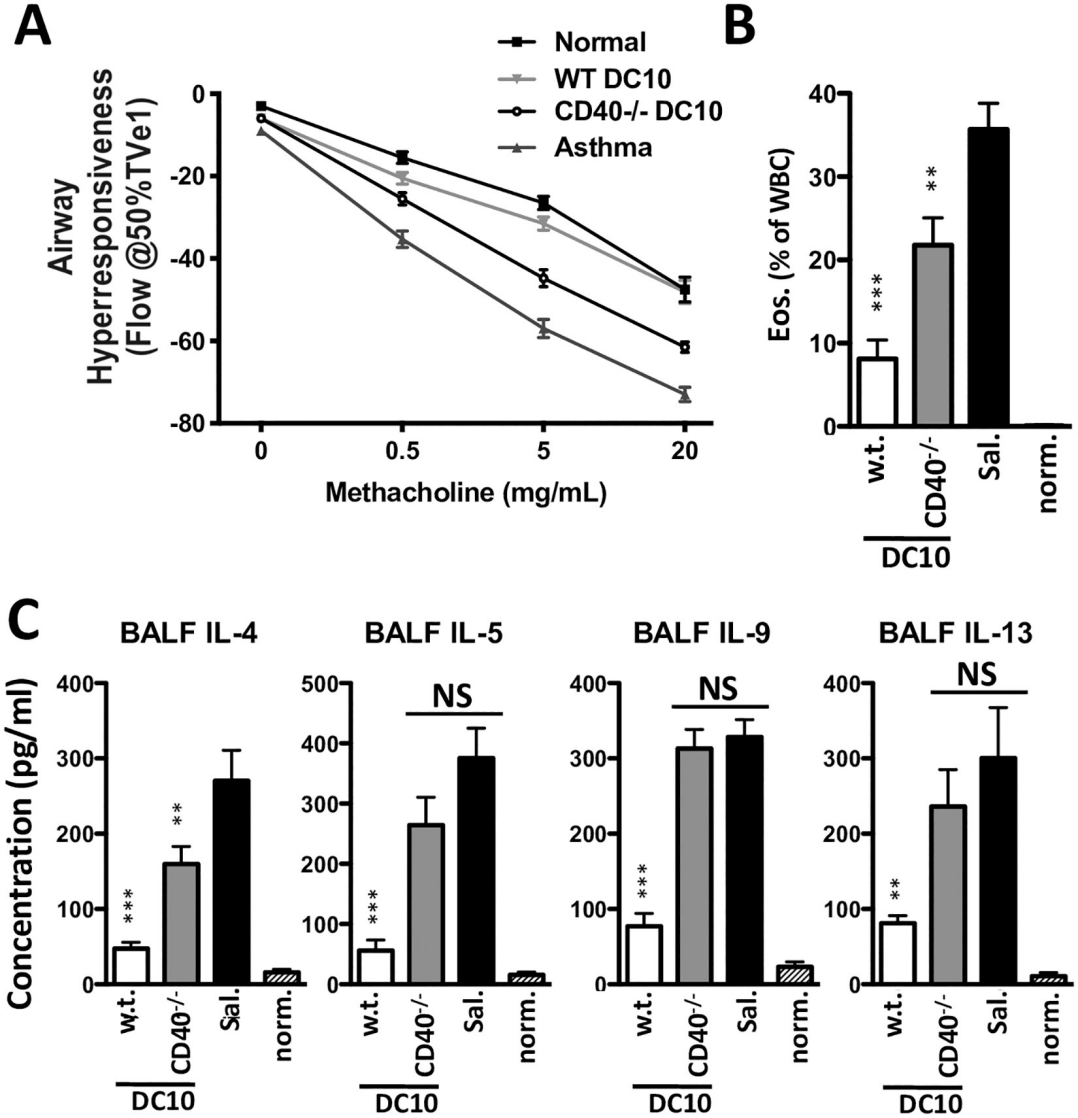
CD40 expression by DC10 is important in their ability to suppress the asthma phenotype. C57BL/6 mice were injected i.p. with 200 μl of OVA-alum (2 μg OVA/mg alum) on days 0 and 14, and then exposed to 1% OVA aerosols for 20 min on days 28, 30 and 32. They were rested for two weeks and then given a single i.p. injection of 1x10^6^ w.t. or CD40^-/-^ OVA-presenting DC10. Control mice included saline-treated asthmatic (Sal.) and healthy normal animals. Four weeks later all mice were again challenged with aerosolized OVA and their asthma phenotype was assessed. **(A)** Airway hyperresponsiveness was determined by head-out plethysmography 24h post allergen exposure. At 48h post allergen exposure mice were sacrificed, and **(B)** BAL eosinophils were enumerated using Wright’s stained cytocentrifuge preparations, while **(C)** BALF Th2 cytokines were determined by ELISA. Bars represent mean with SEM from 9–15 mice from three independent experiments. NS, *, ** or ***, p>0.05, ≤0.05, 0.01 or 0.001, respectively versus CD40^-/-^ DC treatment (AHR) or versus the saline control (all other parameters), as determined using one-way ANOVA tests with Tukey’s *post-hoc* testing.

This lack of regulatory activity by CD40^-/-^ DC10 in our assays did not reconcile well with the noted reports of augmented regulatory activities within CD40-silenced or CD40^-/-^ immature DC in an array of *in vivo* models [14–17, 26]. As such, we next assessed the regulatory activities of our DC10 in our mouse model of OVA-induced asthma, treating asthmatic mice with OVA-pulsed w.t. or CD40^-/-^ DC10 and assessing their asthma phenotype 4 wk later (Fig 2), when the regulatory effects of DC10 have achieved essentially full penetrance, as reported previously [5, 19]. Wild-type DC10 immunotherapy abrogated AHR (p>0.05), and also efficiently suppressed the airway eosinophil and IL-4, IL-5, IL-9 (each, p≤0.001) and IL-13 (p≤0.01) responses to allergen challenge (Fig 2). In keeping with our *in vitro* observations, loss of CD40 expression also markedly inhibited the *in vivo* regulatory activities of DC10. The CD40^-/-^ DC had no significant impact of airway IL-5, IL-9 or IL-13 recall responses to allergen challenge (p>0.05 versus saline-treated asthmatic animals), although they did modestly reduce AHR, and airway eosinophilia and IL-4 (in each case, ≈60% attenuated activity relative to w.t. DC10; Fig 2A, 2B and 2C).

**Fig 3:**
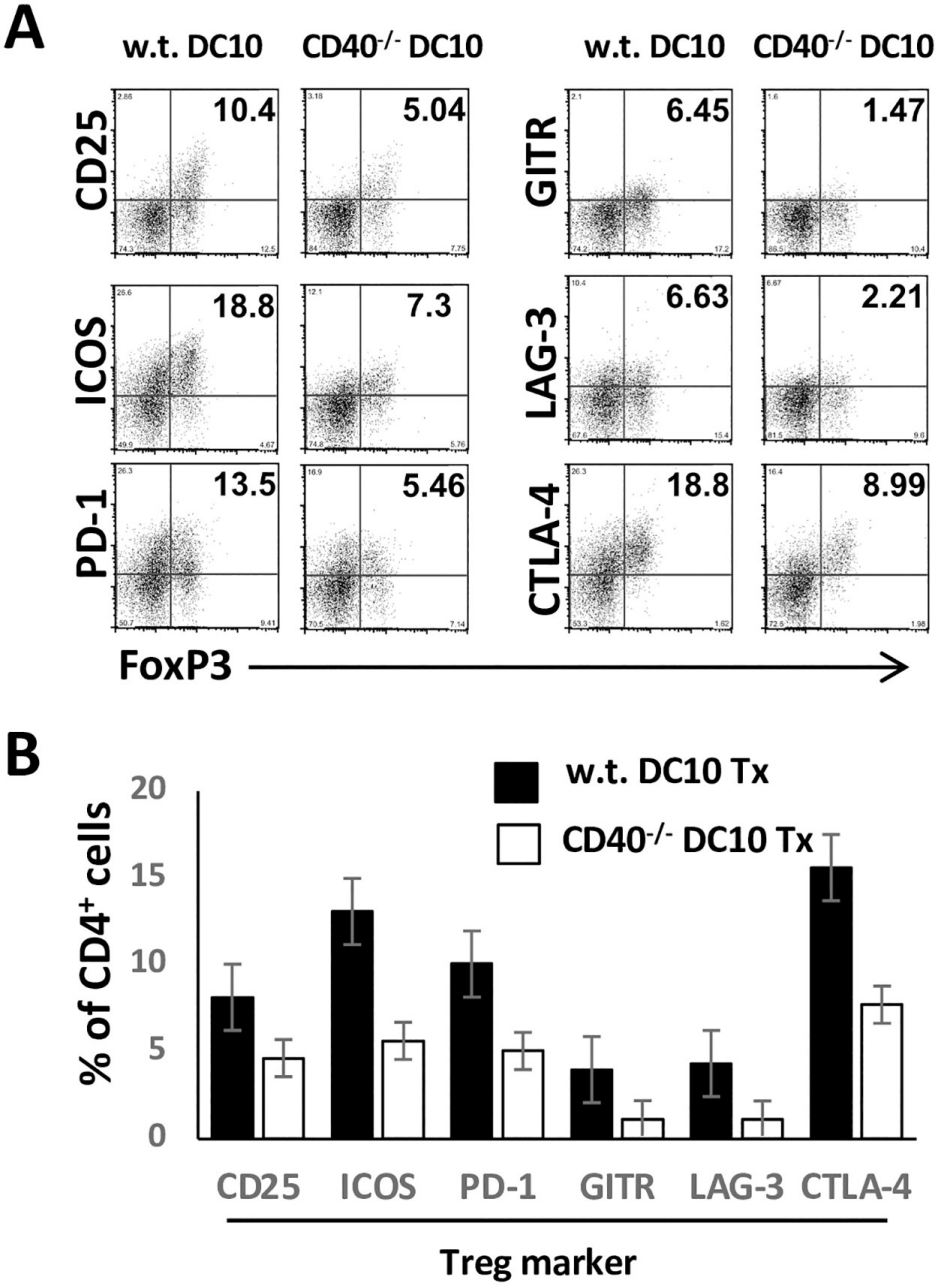
CD40 is important for the induction of Treg in DC10-treated asthmatic mice. Lungs were removed from asthmatic mice treated three weeks earlier with DC10 generated from wild-type (w.t.) and CD40^-/-^ mice, as in Fig 2, and then enzymatically dispersed as in the Materials and Methods. Lung CD4^+^ cells were magnetically sorted, stained for the indicated Treg markers and marker expression was analyzed by flow cytometry. **(A)** Representative scatter plots from one experiment of three undertaken depicting the expression of the indicated markers on the Foxp3^+^/marker-positive CD4^+^ cells. The quadrant boundaries are based on isotype staining, with the percentage of cells in the upper–right quadrant. **(B)**. Graphic presentation of the mean +/- SD of the proportion of the CD4^+^ cells that stained with each marker. This experiment was repeated three times.

DC10 promote tolerance in asthmatic mice by directly suppressing cognate CD4^+^CD25^lo^Foxp3^-^ Th2 phenotype effector T cell responses, but also by inducing these T cells to transdifferentiate into CD25^hi^Foxp3^+^ Treg [8]. Thus, we next magnetically sorted CD4^+^ T cells from the lungs of asthmatic mice treated 3 wk earlier with w.t. or CD40^-/-^ DC10 and assessed the expression of a panel of Treg activation markers. As observed previously [8], the Foxp3^+^ cells from the w.t. DC10-treated mice displayed a robust activation phenotype (Fig 3). There were fewer CD25^+^Foxp3^+^ cells in the lungs of the CD40^-/-^ DC10-treated mice relative to the w.t. DC10 recipients, but also fewer Foxp3^+^ cells that expressed ICOS, PD-1, GITR, LAG3 or CTLA-4 (Fig 3A). Overall, the Treg induced by CD40^-/-^ DC10 expressed these markers at ≈37% of the levels seen with w.t. DC10-induced Treg (Fig 3B). We conclude that CD40 expression on DC10 is important to their suppression of Teff cell responses and to their full induction of activated Treg *in vivo*.

### T cell regulation of cell surface CD40 on DC10

**Fig 4:**
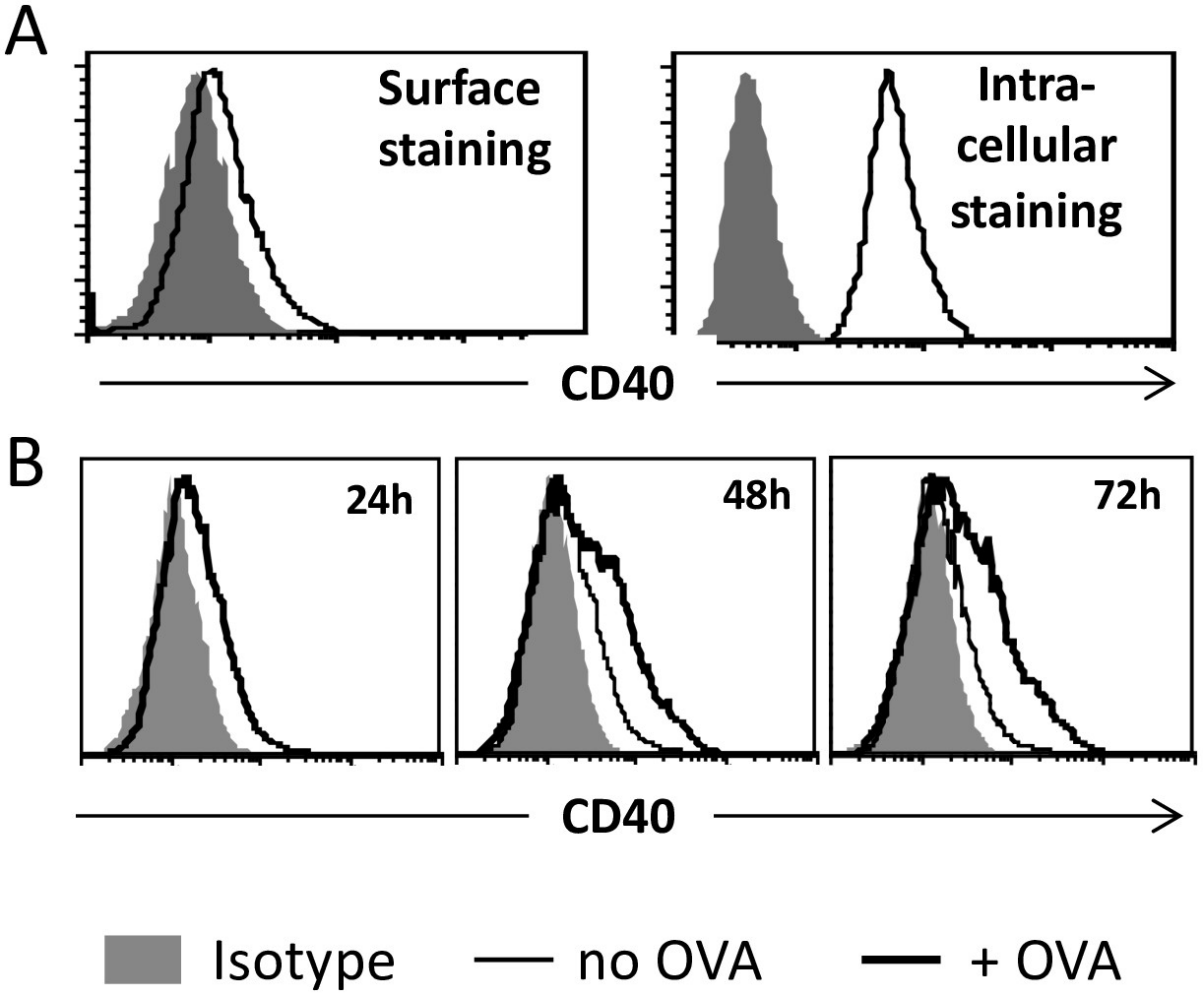
Interaction with cognate T cells leads to upregulation of cell surface CD40 on DC10. DC10 were generated as in Fig 1 and **(A)** stained with anti-CD40 antibody in the absence (cell surface) or presence (intracellular) of a permeabilizing agent and analyzed by flow cytometry. **(B)** OVA-pulsed DC10 (thick line) or DC10 without OVA (thin line, 48 and 72 hr panels) were co-cultured with OVA-specific CD4^+^ cells from D011.10 mice for the indicated times and then their cell surface CD40 was evaluated by flow cytometry. Shaded histograms represent isotype staining. The data presented depict the results of one experiment that is representative of 3–4 independent experiments with duplicate analysis of each sample.

Triggering of CD40 signaling in steady-state DC by T cell CD40L engagement upregulates their expression of CD40, other costimulatory receptors and IL-12, which cumulatively serve to further activate the T cells (e.g., ref. [27]). Numerous reports have documented however that DCreg express lower levels of CD40 and other antigen-presentation-associated costimulatory markers relative to stimulatory DC (reviewed in ref. [4]). This has also been reported for murine and human DC10 [7, 19]. Given that CD40 has been reported to interfere with tolerance induction by DC [12, 13], we assessed whether T cell engagement modulates the levels at which DC10 express CD40. We confirmed by FACS that w.t. DC10 only weakly express CD40 on their cell surface, but also report that they express abundant intracellular CD40 (Fig 4A). However, when cultured for 2–3 days with cognate T cells from OVA-asthmatic donors, OVA-presenting DC10 significantly upregulated their levels of cell surface CD40 (Fig 4B). Indeed, over three experiments the T cell-stimulated DC10 displayed a 200–300 percent increase over isotype control staining in levels of cell surface CD40. This effect was allergen-specific, inasmuch as co-culture of OVA-specific CD4^+^ T cells with DC10 that had not been pulsed with OVA did not impact CD40 expression (Fig 4B; no OVA).

### CD40 ligation augments IL-10, but not IL-12 production by DC10

**Fig 5:**
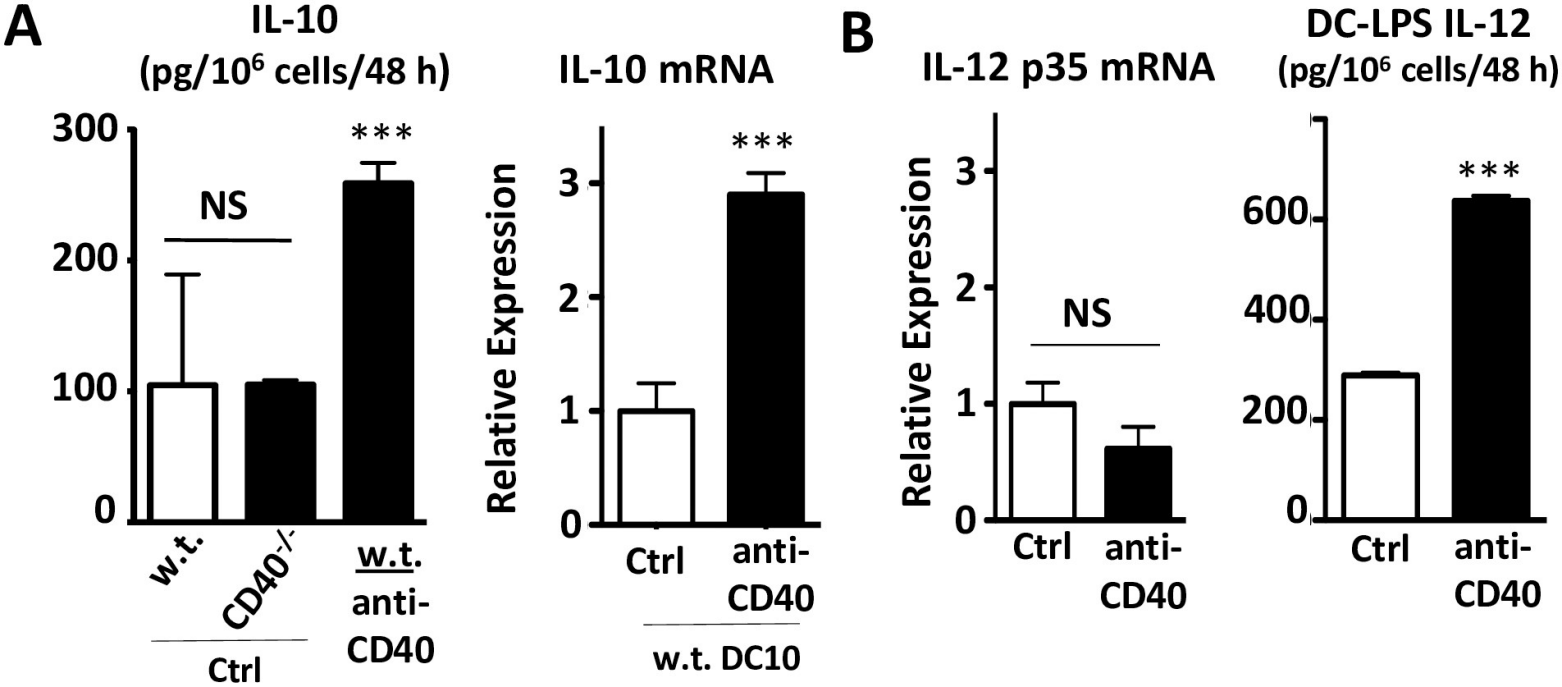
CD40 signaling increases IL-10, but not IL-12 production by DC10. CD40^-/-^ or w.t. DC10 were stimulated with plate-bound hamster IgG1 anti-mouse CD40 or irrelevant hamster IgG1 control antibodies (Ctrl) for two days and expression of **(A)** IL-10 mRNA and **(B, left panel)** IL-12p35 mRNA were determined by qRT-PCR and ELISA. **(B, right panel)** LPS-matured immunostimulatory DC (DC-LPS) were also stimulated with anti-CD40 or irrelevant control antibodies for 48 h, and their expression of IL-12p70 was assessed by ELISA. Bars represent mean with SEM from duplicate or triplicate repetitions from one of three independent experiments. NS or ***, p>0.05 or ≤0.001, respectively versus the w.t. or saline control, as appropriate. For the left hand panel in Fig 5A, we used a one-way ANOVA test with Tukey’s *post-hoc* testing, while students T tests were used for all other panels.

CD40^-/-^ DC10 displayed significantly reduced regulatory activities relative to w.t. DC10, although as steady state cells both populations secrete equally low levels of IL-10 (Fig 5A, Ctrl). This suggests that while IL-10 is critical to the regulatory activity of DC10 [9, 10], basal expression of IL-10 by itself is not sufficient to fully support the cells’ regulatory activities. As noted above (S2 Fig), these cells also express equivalent levels of an array of other DCreg-associated markers, including MHCII, CD80, ICOS-L and PD-L1. While unstimulated w.t. DC10 secreted ≈100 +/- 90 pg of IL-10/10^6^ cells/48 h, exposure to plate-bound anti-CD40 antibody increased IL-10 mRNA levels in these cells by ≈3-fold and IL-10 secretion by ≈2.5-fold. No increase in IL-12 p35 mRNA levels was detected in anti-CD40-treated DC10 (Fig 5A), nor did they secrete detectable levels of IL-12 protein (data not shown). As expected however, anti-CD40 treatment did increase IL-12 production in stimulatory DC-LPS by ≈2.3-fold (Fig 5B). Thus, our data confirms that CD40 signaling by itself does increase IL-10 expression by these DCreg.

### Supplementation of IL-10 expression reconstitutes the regulatory activity of CD40^-/-^ DC10

**Fig 6:**
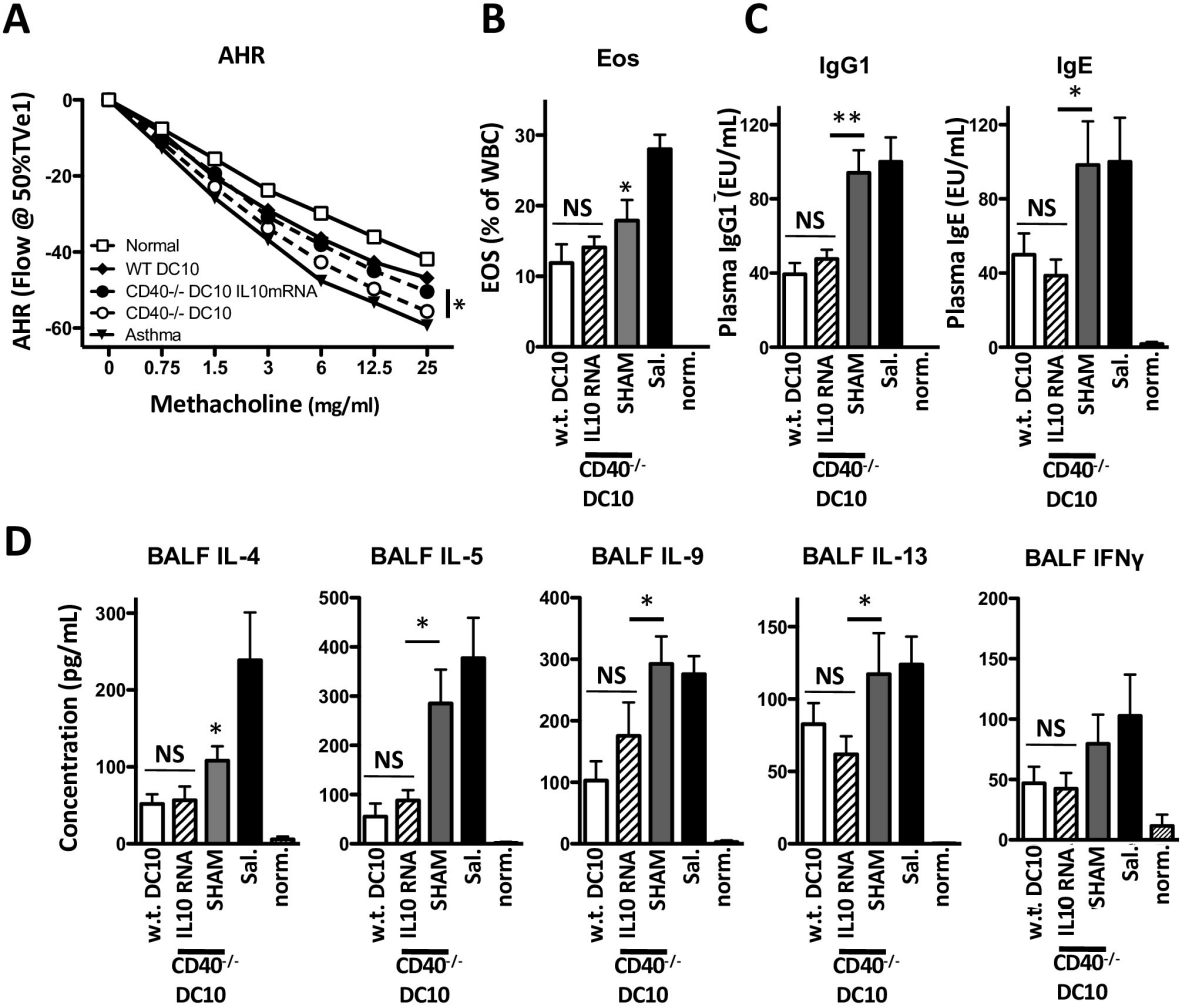
Complementing IL-10 expression in CD40-deficient DC10 restores their ability to suppress the asthma phenotype. Mice were rendered asthmatic as described in Fig 2 and treated with 1x10^6^ w.t. or with CD40^-/-^ DC10 that had been transfected with medium- (SHAM) or IL-10 mRNA-containing (IL-10 RNA) liposomes. Control groups included saline-treated asthmatic mice and normal mice. Four weeks after treatment, mice were challenged again with aerosolized OVA and **(A)** the asthmatic phenotype assessed by measuring AHR, **(B)** BAL eosinophils, **(C)** OVA-specific plasma IgG1 and IgE and **(D)** BALF cytokines. Bars represent mean with SEM from 6–10 mice from two independent experiments. NS, * or **, p>0.05, ≥0.05 or 0.01, respectively versus CD40^-/-^ DC10 treatment (AHR) or versus the saline control (all other parameters), as determined using one-way ANOVA tests with Tukey’s *post-hoc* testing.

The fact that CD40 signaling increased IL-10 expression in DC10 raised the question of whether this is important to the overall regulatory activities of these DCreg. To address this we compared the biological activities of IL-10-reconstituted CD40^-/-^ with w.t. DC10, reconstituting the CD40^-/-^ cells with purified IL-10 mRNA transcribed *in vitro*. Control CD40^-/-^ DC10 were subjected to a ‘sham’ transfection. The IL-10 mRNA transfection increased intracellular IL-10 but not IL-12 p35 mRNA levels, as determined at 48 h after transfection, and led to a substantial increase in IL-10 secretion relative to sham-treated CD40^-/-^ DC10 (S3 Fig). We then used the IL-10-reconstituted or sham-transfected CD40^-/-^ DC10, or w.t. DC10 to treat asthmatic mice and assessed their impact on the asthma phenotype. The w.t. DC10 again induced a robust allergen tolerance, as determined by assessments of AHR, airway eosinophilia and Th2 cytokines, and plasma OVA-specific IgG1 and IgE (Fig 6). Given that asthma can induce a mixed Th1/Th2 phenotype response, we also assessed airway IFNγ levels—the untreated airways contained quite modest levels of IFNγ and DC10 treatment did not affect this in a statistically significant manner (p>0.05; Fig 6D). Overall, the impact of the sham-transfected CD40^-/-^ DC was largely as noted above (Fig 2) for otherwise unmanipulated CD40^-/-^ DC10. On the other hand, IL-10 complementation of our CD40^-/-^ DC10 fully reconstituted the cell’s regulatory activities. There were no statistically significant differences in their efficacy relative to that of w.t. DC10 in terms of correcting AHR, airway eosinophilia or Th2 cytokine responses, or circulating allergen-specific IgE or IgG1 (Fig 6). Taken together, our data indicates that CD40-induced augmentation of IL-10 expression contributed importantly to the regulatory activities of DC10.

## Discussion

Our data shows that the immunoregulatory activities of DC10 were in large part dependent on their expression of CD40. Knocking out CD40 exclusively in these cells led to a degradation of their regulatory activities, culminating in a reduced activation of downstream pulmonary Treg. Prior reports that DC10-induced tolerance is also critically dependent on IL-10 expression by these cells [9, 10] provides circumstantial evidence that suggests a linkage between CD40 signaling and IL-10 secretion. Our data herein provides additional evidence in support of this hypothesis.

It is clear that CD40 expression is essential to a full expression of robust regulatory activities by DC10. This contrasts with prior reports of augmented regulatory activities of CD40-silenced immature bone marrow-derived DC [14–17]. Wild-type DC10 were highly tolerogenic in our mouse model of asthma [5, 19], while CD40^-/-^ DC10 were somewhat compromised in their regulatory activities. We observed that, unlike IL-5, IL-9 or IL-13 levels in the airways of our mice, the levels of IL-4 were modestly lower in the CD40^-/-^ DC10-treated mice relative to animals treated with w.t. DC10—we have no direct evidence regarding a mechanism for a differential effect of CD40^-/-^ DC10 on the IL-4 response. We do know that DC10 given i.p. traverse through the lungs prior to arriving in the lung-draining lymph nodes [9]. We query whether CD40L-expressed on basophils in the airways of our asthmatic mice [28, 29] could be triggered by lung-transiting DC10 to secrete IL-4 [30, 31], which in principle could act to augment overall Th2 sensitization [32]. CD40^-/-^ DC, on the other hand, would have been unable to activate any CD40-dependent IL-4 secretion by basophils as they transit through the lungs, such that these animals could have experienced a modestly reduced level of Th2 sensitization. It has also been reported that OVA challenge induces expansion of group 2 innate lymphoid cells (ILC2) in asthmatic mice [33], and we know that activated airway ILC2 express IL-5, IL-9 and IL-13, but not IL-4 [32]. Our w.t. and CD40^-/-^ DC10-treated mice were both challenged repeatedly with OVA prior to assessment of airway responses, such that both groups would potentially have had elevated numbers of airway ILC2. In principle, that ILC2 response, in conjunction with the ongoing Th2 response, could have contributed to the heightened levels of IL-5, IL-9 and IL-13 we observed in the airways, without a commensurate increase in the IL-4 response.

The fact that unstimulated CD40^-/-^ and w.t. DC10 secreted equally low levels of IL-10 indicates that expression of CD40 in itself is not a prerequisite for IL-10 production, but also that these basal levels of IL-10 expression are not sufficient to fully support the regulatory activities of DC10. CD40 engagement in DC10 by cognate T cells increased their expression of cell surface CD40, which would in principle allow for more intimate DC10-T cell engagement. A T cell CD40L-driven augmentation of IL-10 expression by DC10 could potentially be an immunologic mechanism to channel IL-10 secretion directly onto cognate T cells and thereby focus the DC’s regulatory activities where they are needed most. We propose that it is this augmented CD40-induced production of IL-10 by w.t. DC10 that imparts them with their substantial regulatory phenotype. This is consistent with our observation that the tolerogenic activities of CD40^-/-^ DC10 were fully restored to w.t. DC10 levels by reconstituting the knock-out cells with IL-10 mRNA alone.

It is well recognized that IL-10 expression by DC is a feature of tolerance responses. There have been numerous reports of IL-10 production by DC that have been exposed to CD40 signaling, as well as by DC not so exposed [4]. Indeed, the levels of IL-10 produced by DC cultured with agents such as dexamethasone/vitamin D [34], complement component C1Q [35], vasoactive intestinal peptide/LPS [36], or IL-10/LPS [37] have been equivalent, if not greater than those produced by DC exposed to CD40 signaling [38–40]. Nevertheless, given that the functions of DCreg are realized by virtue of their presenting antigens to T cells, in the end all DCreg that express any level of CD40 would experience CD40L signaling at one level of another. At least some of the reports of high-level IL-10 expression by DC10 that have been cultured with CD40L-transfected cells did not include control DC not exposed to CD40L signaling [38, 39], which complicates interpretation of the data viz-a-viz a role for CD40 signaling in that IL-10 expression. It has been reported that immature human DC substantially increase expression of IL-10 following CD40L stimulation, but these cells also produce 2-3-fold more IL-12 than IL-10 [40]. Interestingly, in the same report the presence of semen, or more specifically E-series prostaglandins therein, CD40L-exposed immature DC then upregulate TGFβ expression and produce more IL-10 than IL-12 [40]. Immature human DC have been noted in one report to secrete negligible IL-10 following CD40L stimulation, although addition of dexamethasone to these cultures modestly increases the IL-10 response [41]. It has also been reported that immature DC do secrete IL-10 following challenge with super-agonistic CD40 trimers, bacteria or TLR agonists [42]. However, in contrast to what might be expected, these latter CD40-triggered DC induced T cells with which they interact to secrete substantially more IFNγ than seen with unstimulated DC [42]. Stimulation of mature human DC with CD40L trimers and IFNγ also induces IL-10 expression, but these cells produce substantially more IL-12 than IL-10, and also induce T cells with which they are cultured to secrete high levels of IFNγ [43]. Thus, based on the published literature the relationship between CD40 signaling and IL-10 expression has not been clear, nor has it been unequivocal that CD40 signaling potentiates the tolerogenicity of DCreg. We have reported that the induction of tolerance by DC10 is critically dependent on their expression of IL-10 and that DC10 given i.p. to asthmatic mice are maximally found in the lungs of the treated mice at +/- 7 dy post-treatment [9]. Allergen-specific T cells are abundant in the lungs of these mice at this time [8], such that engagement DC10 by CD40L on the lung-resident T cells would trigger recruitment of additional CD40 to the plasma membranes in these DC, allowing them to yet more strongly engage with the cognate T cells. We know that DC10 induce CD25^lo^ Th2 Teff cells to transdifferentiate into CD25^hi^Foxp3^+^ Treg and that these Treg begin to appear in the treated mice at ≈2 wk post-treatment [9]. Thus, we hypothesize that this initial CD40-mediated Teff cell engagement of DC10 sets in motion the spiking of IL-10 production needed to convert Teff cells into Treg within the asthmatic animals, and that that is a central facet of their regulatory activities.

As noted above, silencing of CD40 in, or use of CD40^-/-^ immature DC has been reported multiple times to induce tolerance in mouse models of allergic disease [14–17, 26], while we report herein that CD40^-/-^ DC10 are significantly less tolerogenic than w.t. DC10. It is not immediately obvious why our results differ so substantially from the reports of *increased* regulatory activity in CD40-silenced immature DC. Immature DC and DC10 share many features in common, including expressing equivalent levels of CD40, CD54, CD80, CD86 & MHCII [5, 19], equal phagocytic capacities and responsiveness to CCL3 and CCL19, and equal expression of IL-6, IL-12, TGFβ, and IFNγ [6]. However, even steady-state DC10 express substantially more IL-10 than do immature bone marrow-derived DC [6]. This suggests that heightened expression of IL-10 by DC10 could well be a key feature in determining the importance of CD40 signaling to DC regulatory activities. Silencing of CD40 signaling in immature antigen-presenting DC [14, 15, 17, 16], which may secrete IL-10 at levels below that required to support a full-spectrum tolerance response [19], might be expected to lead to T cell anergy, with reduced pathology [14–17]. We found that steady-state DC10 secrete levels of IL-10 equivalent to those produced by CD40^-/-^ DC10 and showed that, while compromised, CD40^-/-^ DC10 still carry some vestige of regulatory activities. Our report that silencing or knocking-out IL-10 expression eliminates the regulatory activities of DC10 [9, 10] supports our hypothesis that CD40-induced expression of IL-10 in DC10 serves the critical function of closely linking IL-10 secretion and T cell:DC10 engagement.

In summary, we report that CD40 signaling in DC10 is critical to these cells realizing the full range of their regulatory functions. That CD40 signaling was important to IL-10 expression and induction of robust tolerance responses suggests that DCreg to be used for immunotherapeutic applications should be designed to take advantage of such CD40-augmented regulatory activities. We observed that w.t. and CD40^-/-^ DC10 express equal basal levels of IL-10, that cognate T cells induce DCreg to upregulate their cell surface CD40, and that signaling through that CD40 augments expression of IL-10 by 2-3-fold. This further suggests that, as noted, T cell engagement of DCreg CD40, with consequent upregulation of their IL-10 response may be a central mechanism for simultaneously augmenting this cells’ regulatory activities and focusing them directly onto their T cell targets.

## Supporting information

S1 Fig FACS analysis of asthmatic lung T cells purified by magnetic sorting with CD4-specific paramagnetic beads. Single cell suspensions of enzymatically dispersed cells from the lungs of asthmatic mice were labelled with CD4-specific paramagnetic beads and run through magnetic sorting columns. The cells retained on the columns were eluted, washed and assessed for expression of CD4 by FACS staining with fluorochrome-labelled CD4-specific (white histogram) or isotype control (grey histogram) antibodies.
(PDF)

S2 Fig Comparison of cell surface marker expression on DC10 generated from wild type or CD40^-/-^ mice. DC10 were generated from w.t. or CD40^-/-^ mice and the expression of the indicated cell surface receptors was determined by flow cytometry. Solid and dashed line histograms represent WT or CD40^-/-^ DC10, respectively, and shaded histograms isotype-matched control. The data presented are from one representative experiment of two undertaken.
(PDF)

S3 Fig Expression of IL-10 in CD40^-/-^ DC10 transfected with IL-10 mRNA or medium-containing liposomes. DC10 generated from CD40^-/-^ mice were transfected with IL-10 mRNA (IL-10) or subjected to a sham transfection protocol (SHAM) as in Fig 6. Relative expression of IL-10 mRNA and protein and IL-12p35 mRNA were determined by qRT-PCR. Secreted IL-10 was quantified by ELISA 24 h and 48 h after transfection. The data presented are from one representative experiment of two undertaken.
(PDF)

S4 Fig Raw data for Fig 4. (ZIP)

S6 File (JO)

S7 File (JO)

S8 File (XLSX)

S9 File (PNG)
