# Supplementary material for: CD40 signaling augments IL-10 expression and the tolerogenicity of IL-10-induced regulatory dendritic cells
Source: PLoS One. 2021 Apr 1;16(4):e0248290. doi: 10.1371/journal.pone.0248290 (PMC8016274; doi:10.1371/journal.pone.0248290)
Supplement: S1 Fig — Single cell suspensions of enzymatically dispersed cells from the lungs of asthmatic mice were labelled with CD4-specific paramagnetic beads and run through magnetic sorting columns. The cells retained on the columns were eluted, washed and assessed for expression of CD4 by FACS staining with fluorochrome-labelled CD4-specific (white histogram) or isotype control (grey histogram) antibodies. (PDF) [file pone.0248290.s001.pdf]

Dawicki et al, Suppl. Fig. S1

CD4+ T cell FACS

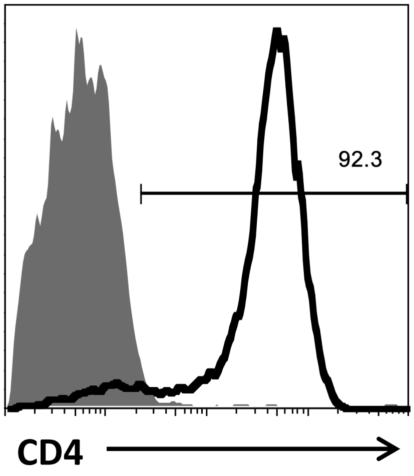

Supplementary Figure S1. FACS analysis of asthmatic lung T cells purified using magnetic sorting with CD4-specific paramagnetic beads. Single cell suspensions of enzymatically-dispersed cells from the lungs of asthmatic mice were labelled with CD4-specific paramagnetic beads and run through magnetic sorting columns. The cells retained on the columns were eluted, washed and assessed for expression of CD4 by FACS staining with fluorochrome-labelled CD4-specific (white histogram) or isotype control (grey histogram) antibodies.
