# Supplementary material for: CD40 signaling augments IL-10 expression and the tolerogenicity of IL-10-induced regulatory dendritic cells
Source: PLoS One. 2021 Apr 1;16(4):e0248290. doi: 10.1371/journal.pone.0248290 (PMC8016274; doi:10.1371/journal.pone.0248290)
Supplement: S2 Fig — DC10 were generated from w.t. or CD40-/- mice and the expression of the indicated cell surface receptors was determined by flow cytometry. Solid and dashed line histograms represent WT or CD40-/- DC10, respectively, and shaded histograms isotype-matched control. The data presented are from one representative experiment of two undertaken. (PDF) [file pone.0248290.s002.pdf]

Dawicki et al, Suppl. Fig. S2

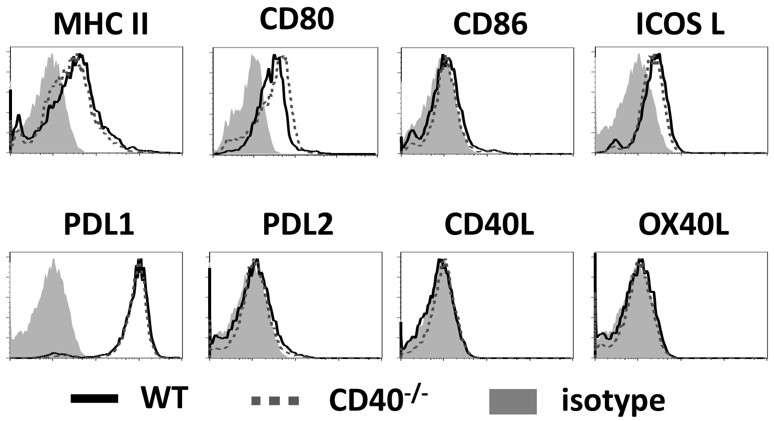

Supplementary Figure S2. Comparison of cell surface marker expression on DC10 generated from wild type or CD40$^{-/-}$ mice. DC10 were generated from w.t. or CD40$^{-/-}$ mice and the expression of the indicated cell surface receptors was determined by flow cytometry. Solid and dashed line histograms represent WT or CD40$^{-/-}$ DC10, respectively, and shaded histograms isotype-matched control. The data presented are from one representative experiment of two undertaken.
