# Supplementary material for: CD40 signaling augments IL-10 expression and the tolerogenicity of IL-10-induced regulatory dendritic cells
Source: PLoS One. 2021 Apr 1;16(4):e0248290. doi: 10.1371/journal.pone.0248290 (PMC8016274; doi:10.1371/journal.pone.0248290)
Supplement: S3 Fig — DC10 generated from CD40-/- mice were transfected with IL-10 mRNA (IL-10) or subjected to a sham transfection protocol (SHAM) as in Fig 6. Relative expression of IL-10 mRNA and protein and IL-12p35 mRNA were determined by qRT-PCR. Secreted IL-10 was quantified by ELISA 24 h and 48 h after transfection. The data presented are from one representative experiment of two undertaken. (PDF) [file pone.0248290.s003.pdf]

Dawicki et al, Suppl. Fig. S3

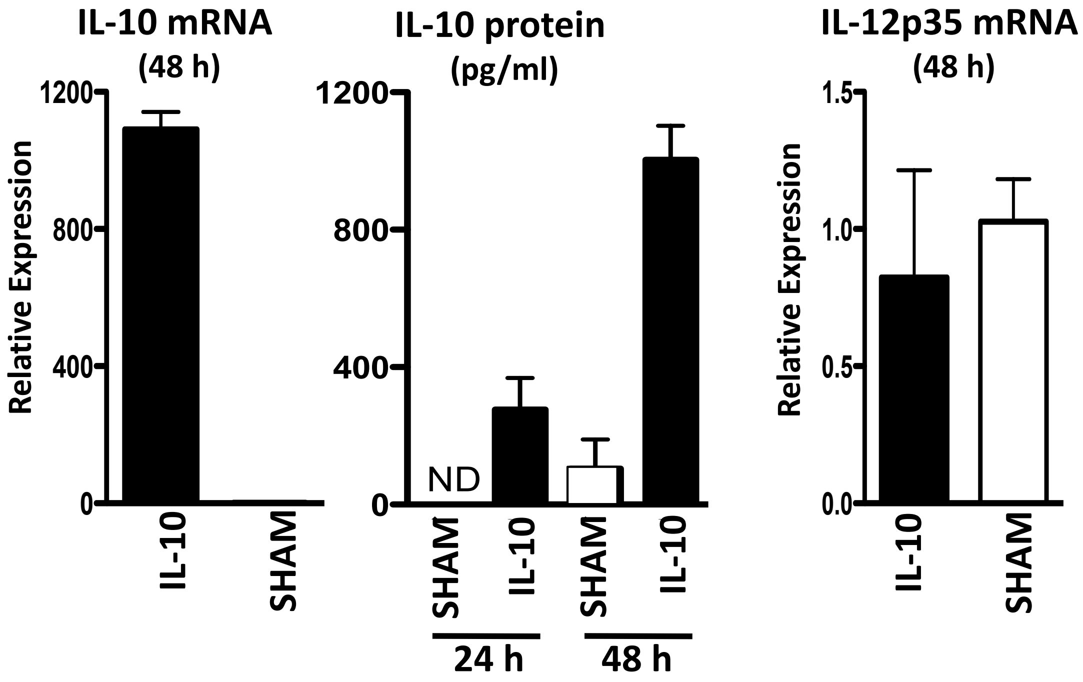

Supplementary Figure S3. Expression of IL-10 in CD40$^{-/-}$ DC10 transfected with IL-10 mRNA or medium-containing liposomes. DC10 generated from CD40$^{-/-}$ mice were transfected with IL-10 mRNA (IL-10) or subjected to a sham transfection protocol (SHAM) as in Fig 6. Relative expression of IL-10 mRNA and protein, and IL-12p35 mRNA were determined by qRT-PCR. Secreted IL-10 was quantified by ELISA 24 h and 48 h after transfection. Bars represent mean +/- SEM from one representative experiment of two undertaken.
