# Supplementary figures and images for: CD40 signaling augments IL-10 expression and the tolerogenicity of IL-10-induced regulatory dendritic cells
Source: PLoS One. 2021 Apr 1;16(4):e0248290. doi: 10.1371/journal.pone.0248290 (PMC8016274; doi:10.1371/journal.pone.0248290)

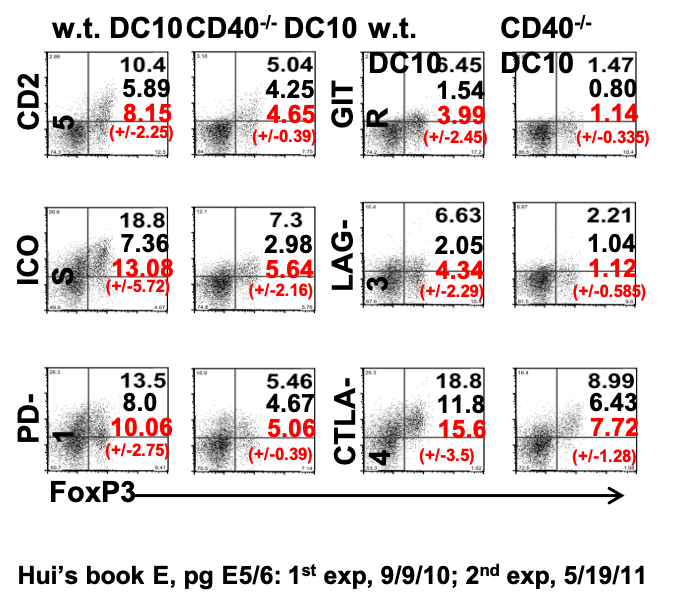

Supplement: S9 File — (PNG) [file pone.0248290.s008.png]
